# Participation in Fraternity and Sorority Activities and the Spread of COVID-19 Among Residential University Communities — Arkansas, August 21–September 5, 2020

**DOI:** 10.15585/mmwr.mm7001a5

**Published:** 2021-01-08

**Authors:** Kristyn E. Vang, Elisabeth R. Krow-Lucal, Allison E. James, Michael J. Cima, Atul Kothari, Namvar Zohoori, Austin Porter, Ellsworth M. Campbell

**Affiliations:** ^1^Arkansas Department of Health; ^2^Office of the Deputy Director for Public Health Service and Implementation Science, Global Immunization Division, Center for Global Health, CDC; ^3^Epidemic Intelligence Service, CDC; ^4^Department of Epidemiology, Fay W. Boozman College of Public Health, University of Arkansas for Medical Science, Little Rock, Arkansas; ^5^Department of Health Policy and Management, Fay W. Boozman College of Public Health, University of Arkansas for Medical Sciences, Little Rock, Arkansas; ^6^Division of HIV/AIDS Prevention, National Center for HIV/AIDS, Viral Hepatitis, STD, and TB Prevention, CDC.

Preventing transmission of SARS-CoV-2, the virus that causes coronavirus disease 2019 (COVID-19), in colleges and universities requires mitigation strategies that address on- and off-campus congregate living settings as well as extracurricular activities and other social gatherings (*1*–*4*). At the start of the academic year, sorority and fraternity organizations host a series of recruitment activities known as rush week; rush week culminates with bid day, when selections are announced. At university A in Arkansas, sorority rush week (for women) was held during August 17–22, 2020, and consisted of on- and off-campus social gatherings, including an outdoor bid day event on August 22. Fraternity rush week (for men) occurred during August 27–31, with bid day scheduled for September 5. During August 22–September 5, university A–associated COVID-19 cases were reported to the Arkansas Department of Health (ADH). A total of 965 confirmed and probable COVID-19 cases associated with university A were identified, with symptom onset occurring during August 20–September 5, 2020; 31% of the patients with these cases reported involvement in any fraternity or sorority activity. Network analysis identified 54 gatherings among all linkages of cases to places of residence and cases to events, 49 (91%) were linked by participation in fraternity and sorority activities accounting for 42 (72%) links among gatherings. On September 4, university A banned gatherings of ≥10 persons, and fraternity bid day was held virtually. The rapid increase in COVID-19 cases was likely facilitated by on- and off-campus congregate living settings and activities, and health departments should work together with student organizations and university leadership to ensure compliance with mitigation measures.

University A began the academic year on August 24, offering in-class and virtual instruction for approximately 20,000 students; a majority of students used virtual instruction. Before and during the start of the academic year, students might have participated in on- and off-campus fraternity or sorority activities. Cases were identified by the university during an ADH-sponsored testing event (September 1–3) or linked to the university by ADH. Case data were reported to ADH by clinic staff members at university A and stored in an electronic database (REDCap, version 8.8.0; Vanderbilt University). Using a standardized questionnaire, ADH nurses interviewed persons with university-associated COVID-19 to ascertain demographic characteristics, date of symptom onset, university status, class attendance type (in-class or virtual), place of residence, and involvement in community- and school-related activities during the 14 days preceding illness onset. Illness onset was defined as the earliest date of reported symptoms for symptomatic cases* or as date of specimen collection for asymptomatic cases (ADH followed up with cases that had testing and diagnosis before symptom onset to identify date of symptom onset). Initial case reviews by ADH identified patients who reported recent participation in sorority or fraternity activities; subsequent infections were observed among cohabitants of these persons.

A network analysis was performed to assess the relationship between participation in university fraternity or sorority activities and the spread of COVID-19 among residential communities at university A. MicrobeTrace, a network analysis and visualization tool developed by CDC,^†^ was used to visualize and describe the full network of persons with COVID-19 and identify potential transmission-related gatherings (network analysis terminology would refer to these as “communities”), defined as one or more gatherings in which multiple cases are identified and epidemiologically linked. COVID-19 patients were included in the network if they lived on campus, participated in fraternity or sorority activities, or lived in the same off-campus dwelling as a person who participated in these activities. Case-to-residence and case-to-event networks were constructed to map residential university A transmission-related gatherings. These networks were used to infer gatherings by algorithmically partitioning the network into the most densely linked cases (nodes) (*5*,*6*). In this network analysis method, the algorithm randomly assigns nodes a “community” identifier, and community connectivity is measured. For each person in the network, community identifiers are swapped with their neighbors and then remeasured. Only changes in community affiliation that improve connectivity are preserved, reversing any swaps that do not improve connectivity. This activity was reviewed by CDC and was conducted consistent with applicable federal law and CDC policy.^§^

During August 22–September 5, a total of 965 university-associated COVID-19 cases with illness onset on or after August 20 were identified, including 699 (72%) confirmed cases (with positive reverse transcription–polymerase chain reaction test results) and 266 (28%) probable cases (with positive results for SARS-CoV-2 antigen test, performed at the university clinic). A 3-day, ADH-sponsored testing event, held during September 1–3 in response to an increase in university-associated cases detected by ADH, resulted in a 22% test positivity rate and identified 324 cases; overall, 34% of cases were identified through this testing event. Among the 965 confirmed and probable cases with illness onset dates during August 20–September 5, 673 (70%) occurred in women and 936 (97%) were in persons aged 18–24 years (Table). Five cases (<1%) in persons identified as faculty or staff members were reported. The number of cases with reported illness onset on a given day increased and peaked on August 31 (Figure 1). Forty-eight (5%) persons with COVID-19 had received in-class instruction, 292 (31%) participated in fraternity or sorority activities, and 149 (15%) lived in a fraternity or sorority house.

**TABLE:** Characteristics of COVID-19 cases associated with university A –– Arkansas, August 20–September 5, 2020

Characteristic	No. (%) of cases
**Total**	**965 (100)**
**Sex**	
Women	673 (70)
Men	234 (24)
Unknown	58 (6)
**University status**	
Student	761 (79)
Faculty/Staff member	5 (0.5)
Other/Unknown	199 (21)
**Age group, yrs**	
0–17	7 (1)
18–24	936 (97)
25–44	18 (2)
45–64	2 (0.2)
≥65	0 (—)
Unknown	2 (0.2)
**Fraternity/Sorority activity participation**	
Any	292 (31)
None	497 (52)
Unknown	176 (18)
**Class attendance type**	
In-class instruction only	48 (5)
Virtual instruction only	453 (47)
Mixed (in-class and virtual)	228 (24)
Unknown	236 (24)
**Housing type**	
On-campus (dormitory)	199 (21)
Off-campus apartment/house	499 (52)
Off-campus fraternity/sorority	149 (15)
Unknown	118 (12)

**Abbreviation:** COVID-19 = coronavirus disease 2019.

**FIGURE 1:**
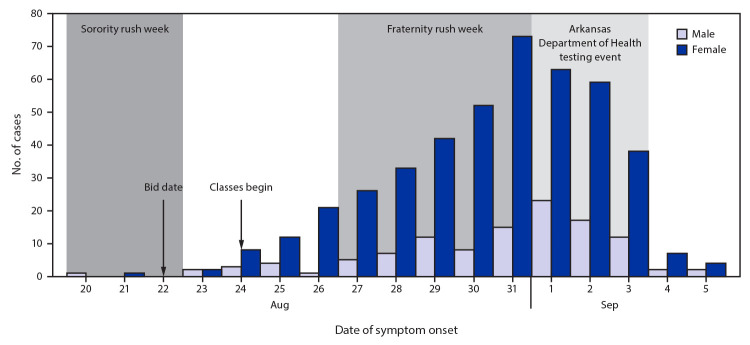
University-affiliated COVID-19 cases (N = 555),* by symptom onset date^†^ and sex — Arkansas, August 17–September 5, 2020^§^ **Abbreviation:** COVID-19 = coronavirus disease 2019. * Ten cases with onset before sorority rush week, which occurred at a frequency of ≤1 per day, are not included. ^†^ The case report date is used as the symptom onset date for asymptomatic cases. ^§^ Bid date refers to date when recruitment decisions were announced.

The network analysis linked 565 (59%) cases to 56 residences (16 dormitories, 20 apartments and houses, and 20 fraternity or sorority houses) and to their case-to-event associations (Figure 2). The full network consisted of one large, linked cluster of 471 (83%) cases (86% in women and 14% in men) and eight smaller, unlinked gatherings of 94 total cases (49% in women and 51% in men; cluster size range = 4–12 cases). Fifty-four gatherings were detected, including 27 (50%) with at least five cases. Among persons in 44 (81%) gatherings, at least one member regularly attended in-person classes, and at least one member of each of the 49 gatherings (91%) reported participation in fraternity or sorority activities or events. Gatherings included an average of 20.3 cases (median = 21; range = 5–44). Among 58 links between gatherings, 42 (72%) were associated with fraternity or sorority activities, 11 (19%) with on-campus dormitories, and five (9%) with off-campus apartments and houses.

**FIGURE 2:**
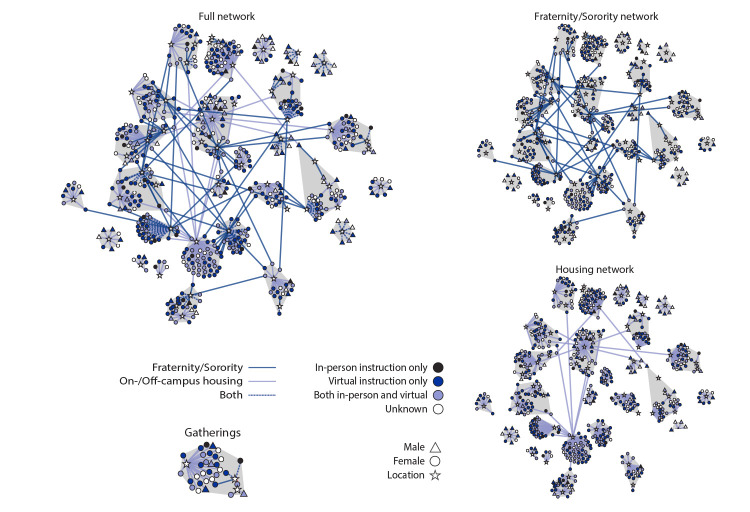
Network diagram of 565 university-affiliated COVID-19 cases connected to the patient’s place of residence or fraternity or sorority participation — Arkansas, August 20–September 5, 2020 **Abbreviation:** COVID-19 = coronavirus disease 2019.

## Discussion

Within 2 weeks of the start of the 2020–21 academic year, COVID-19 cases rapidly increased among persons associated with university A. Transmission was likely facilitated by on- and off-campus congregate living settings and activities, with a majority of the gatherings (91%) and links between them (72%) associated with fraternities or sororities. Most patients reported virtual instruction only, which indicates transmission likely occurred primarily outside the classroom; this finding is supported by the very small proportion of cases among faculty and staff members (0.5%). Women constitute 54% of university A’s 2020 student body but accounted for 70% of university A’s COVID-19 cases. Among linked gatherings, women accounted for 86% of cases, a finding that could reflect involvement in gender-specific activities, including sorority rush week, which held an in-person outdoor bid day event and occurred before fraternity rush week, which was both held later and virtually.

Understanding networks can provide insights into COVID-19 transmission dynamics and inform effective mitigation strategies. In the absence of detailed person-to-person transmission data from contact tracing, network analysis using available data on place of residence and involvement in on- and off-campus activities was used to describe university A’s transmission network, potential gatherings where transmission might have taken place, and links between nodes. The network visualization tool depicted algorithm-detected gatherings to identify links indicating likely recent contact. Visualized in real-time, information from such links and networks could support implementation of targeted mitigation activities, such as isolation of cases and quarantining of contacts.

The findings in this report are subject to at least four limitations. First, incomplete case investigations resulted in missing or unknown data and exclusion from the network analysis. Second, housing and event attendance might not approximate transmission histories. Third, many cases were identified during mass testing events, and event advertisement and location could have resulted in a biased sample. Finally, data were not collected from uninfected persons or on adherence to mitigation strategies, such as social distancing, mask use, and hand hygiene.

Because of the potential for rapid transmission of SARS-CoV-2 in on- and off-campus university settings, student organizations could help ensure compliance with CDC-recommended COVID-19 mitigation measures, such as limiting the size of social gatherings, adhering to social distancing recommendations, requiring mask use, improving hand hygiene, and increasing testing. Encouraging more virtual activities, including those related to fraternity and sorority rush week, might help minimize the risk for transmission on university and college campuses. To ensure compliance with mitigation measures, health departments should work together with student organizations and university leaders.

**Summary**

**What is already known about this topic?**

Preventing COVID-19 in colleges and universities requires mitigation strategies addressing on- and off-campus congregate living settings, extracurricular activities, and social gatherings.

**What is added by this report?**

At the start of the 2020–21 academic year, COVID-19 cases increased rapidly at an Arkansas university. Network analysis indicated that 91% of gatherings were associated with fraternity or sorority activities. Recruitment events held virtually were associated with fewer cases than those held in-person.

**What are the implications for public health practice?**

Given the potential for rapid SARS-CoV-2 transmission in on- and off-campus settings and activities, colleges and universities should work with local health departments and student organizations to ensure compliance with mitigation guidelines.

## Footnotes

* ADH followed up with cases that had testing and diagnosis before symptom onset to identify the actual date of symptom onset.

† https://www.biorxiv.org/content/10.1101/2020.07.22.216275v1.

§ 45 C.F.R. part 46.102(l)(2), 21 C.F.R. part 56; 42 U.S.C. Sect. 241(d); 5 U.S.C. Sect. 552a; 44 U.S.C. Sect. 3501 et seq.
